# Emerging roles of platelet concentrates and platelet-derived extracellular vesicles in regenerative periodontology and implant dentistry

**DOI:** 10.1063/5.0099872

**Published:** 2022-09-01

**Authors:** Jiayue Sun, Yinghan Hu, Yinxin Fu, Derong Zou, Jiayu Lu, Chengqi Lyu

**Affiliations:** 1 Department of Stomatology, Shanghai Jiao Tong University Affiliated Sixth People’s Hospital, Shanghai 200233, China; 2 Wuhan Fourth Hospital, Wuhan, Hubei 430032, China

## Abstract

Platelet concentrates (PCs) are easily obtained from autogenous whole blood after centrifugation and have evolved through three generations of development to include platelet-rich plasma, platelet-rich fibrin, and concentrated growth factor. Currently, PCs are widely used for sinus floor elevation, alveolar ridge preservation, periodontal bone defects, guided bone regeneration, and treatment of gingival recession. More recently, PCs have been leveraged for tissue regeneration to promote oral soft and hard tissue regeneration in implant dentistry and regenerative periodontology. PCs are ideal for this purpose because they have a high concentration of platelets, growth factors, and cytokines. Platelets have been shown to release extracellular vesicles (P-EVs), which are thought to be essential for PC-induced tissue regeneration. This study reviewed the clinical application of PCs and P-EVs for implant surgery and periodontal tissue regeneration.

## I. INTRODUCTION

An increasing number of people worldwide are becoming concerned about their periodontal health. Patients with severe problems are increasingly opting for treatment with oral implants to repair dentition defects and rehabilitate edentulous jaws. Possessing adequate alveolar bone in all three cardinal axes is the basic requirement for implant placement.^1^ Possessing sufficient quality and quantity of soft tissue is an effective guarantee of achieving long-term health of the implant.^2,3^ Artificial bone materials, soft tissue flap grafts, and autologous platelet concentrate (PC) products are now being used routinely to overcome the challenge of sufficiently augmenting soft and hard tissue for regenerative periodontology and implant dentistry.^4^

Platelets are an important component in the regulation of coagulation and hemostasis.^5,6^ A growing number of studies have demonstrated that platelets can also act as regulators in angiogenesis and tissue regeneration. Platelets are relatively easy to obtain for experimental or clinical purposes. Using differences in settling velocity of blood constituents, large numbers of platelets from autologous whole blood can be collected by differential centrifugation to obtain PCs.^7^ PC collection and separation techniques have been refined over the years, and researchers now can isolate three generations or main types of concentrates: platelet-rich plasma (PRP), platelet-rich fibrin (PRF), and concentrated growth factor (CGF). In addition to being rich in platelets, some types of PCs also contain fibrin and leukocytes. More importantly, many bioactive factors are released when PCs are applied topically for tissue repair. These releases include growth factors, cytokines, lysosomes, and adhesion proteins that initiate a signaling cascade, resulting in the binding to corresponding receptors, intracellular biochemical changes, and promotion of regeneration.^8,9^

**FIG. 1.**
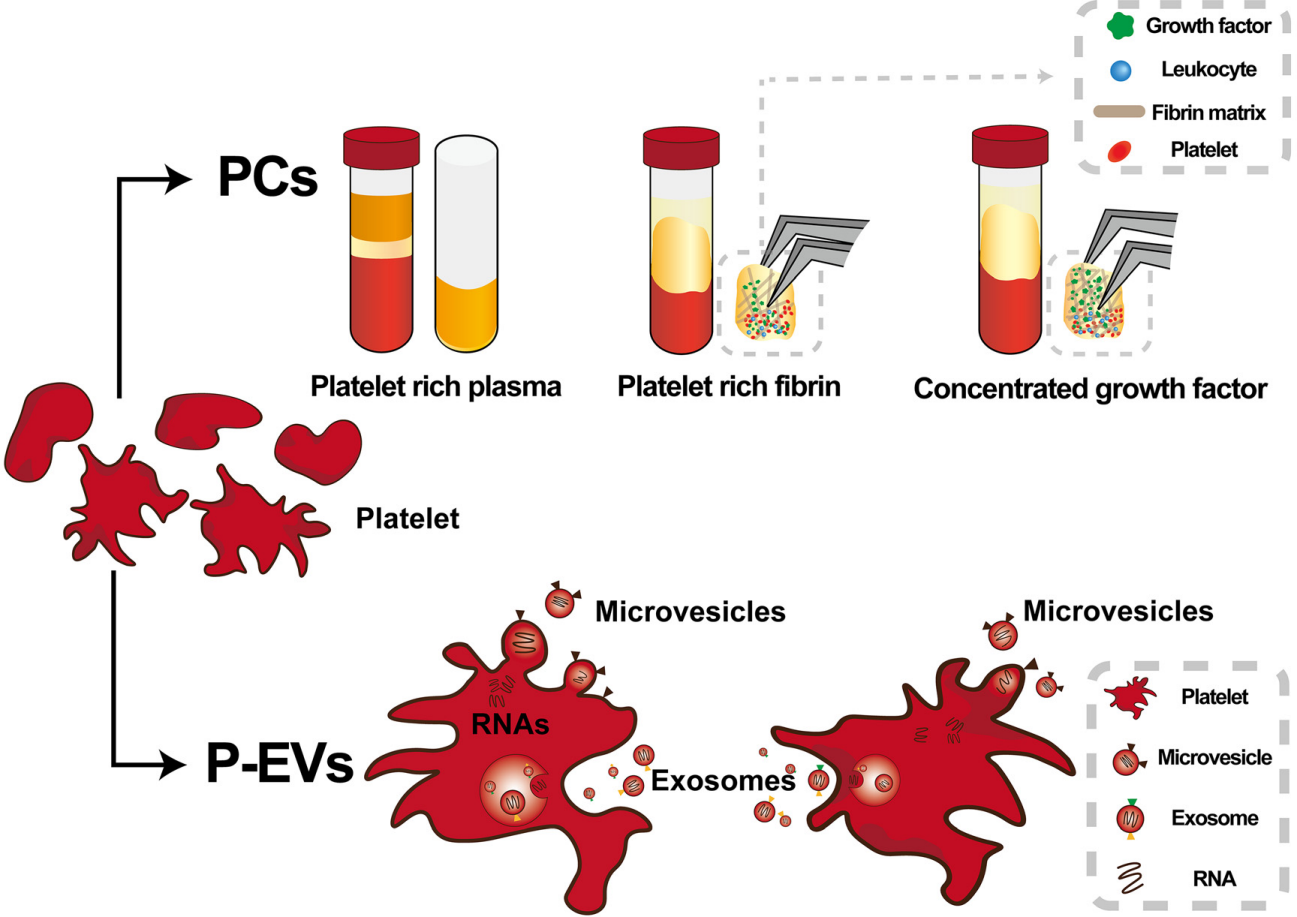
Schematic diagram of platelet concentrates (PCs) and platelet-derived extracellular vesicles (P-EVs).

In recent years, the importance of extracellular vesicles (EVs) in intercellular communication has become more appreciated and understood. EVs can alter the phenotype and function of recipient cells by delivering various proteins, bioactive lipids, and even genetic information.^10^ Platelets can be released into the bloodstream during thrombosis. Upon activation, in addition to chemokines and cytokines, platelets release two different kinds of EVs—exosomes (EXOs) and microvesicles collectively referred to as platelet-derived extracellular vesicles (P-EVs). Currently, P-EVs are used for treating various pathophysiological conditions such as wound healing, inflammatory response, angiogenesis, and neuroregenerative response (Fig. 1).^11–13^

## II. PLATELET CONCENTRATES (PCS) AND PLATELET-DERIVED EXTRACELLULAR VESICLES (P-EVS)

### A. Preparation of platelet concentrates (PCs)

PCs are obtained by gradient centrifugation of autologous venous blood and have shown positive results for tissue regeneration in periodontology and implant dentistry (Fig. 1). However, there is no international consensus on the centrifugal speed, centrifugal force, centrifugal time, and preparation temperature for the preparation of PC. Aside from that, some researchers have defined the preparation parameters in terms of rotational speed (rpm), while others have described them in terms of centrifugal force (g). Centrifugation parameters and several other procedural variables can affect efficacy and reproducibility. Each type of PC has its own sensitivities to preparation variables. PRP requires two tedious centrifugation steps, PRF requires only one step, and CGF preparation requires differential centrifugation.

#### 1. Platelet-rich plasma (PRP)

**FIG. 2.**
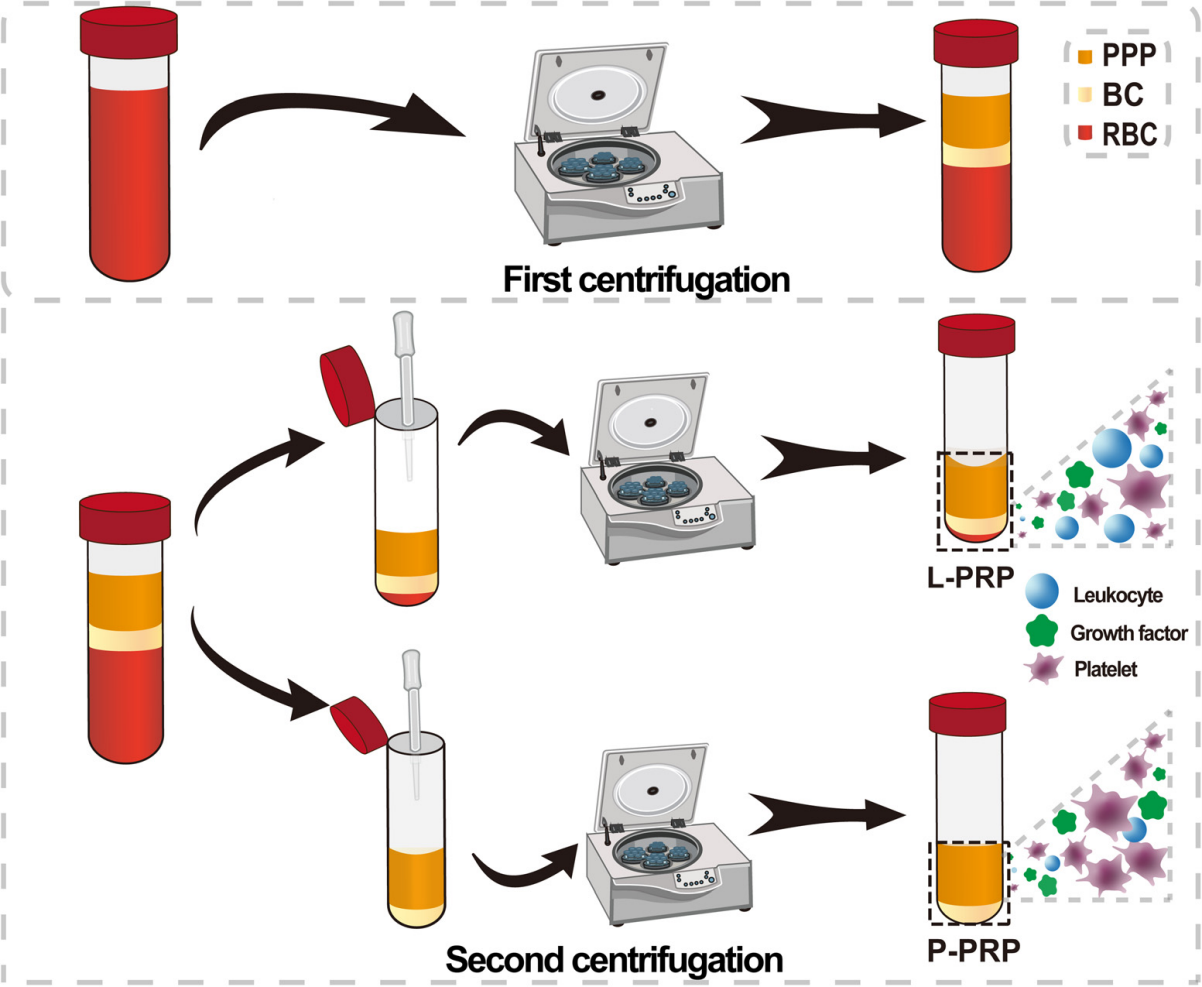
Schematic diagram of the preparation process of different types of PRP (L-PRP and P-PRP).

PRP is a first-generation PC, originally developed and refined in the 1980s. Normally PRP is obtained in liquid form through a tedious two-step centrifugation process. There is still no standard for the best protocol for PRP preparation. Regardless of the speed and time of centrifugation, three layers are obtained in this first gentle centrifugation step. This spin with low acceleration force and short spin time produces, from bottom to top, a red blood cell (RBC) layer, a buffy coat (BC) layer containing leukocytes and platelets, and a platelet-poor plasma (PPP) layer. At this step, PRP is processed differently to obtain either leucocyte- and platelet-rich plasma (L-PRP) or pure platelet-rich plasma (P-PRP) (Fig. 2). The main difference between L-PRP and P-PRP is that their leucocyte content differs.^7^ To obtain L-PRP, after first centrifugation at 160 g for 10 min, the PPP, BC layers, and some residual RBCs’ layer are carefully aspirated out of the centrifuge tube and centrifuged at 250 g for 15 min again.^14^ The resulting supernatant is mostly discarded, leaving a small amount of bottom sediment to obtain L-PRP. L-PRP contains platelets, growth factors, and leukocytes. P-PRP is obtained only by centrifugation of the original PPP layer and superficial BC layer and contains almost no leukocytes. Yin explored six centrifugation protocols and found that P-PRP obtained by first centrifugation at 160 g for 10 min and second centrifugation at 250 g for 15 min had the highest enrichment of platelets and growth factors.^15^

L-PRP and P-PRP can be activated to a gel state by adding calcium chloride solution, thrombin, collagen, or calcium gluconate. When procoagulants are added to activate the PRP solution into a gel, these procoagulants interact with pre-existing anticoagulants, and platelets are activated to release fibrinogen, which polymerizes into fibrin and connects to a mesh, forming a gel-like substance with some adhesion and strength. They can also be transformed into a gel via lysis by freezing.

#### 2. Platelet-rich fibrin (PRF)

Obtaining PRF involves a simple one-step process: whole blood is collected in glass centrifuge tubes and centrifuged. As no anticoagulant is added, the whole blood coagulates naturally during centrifugation. Centrifugation produces an RBC layer at the bottom of the tube, followed by a PRF layer in the middle and a PPP layer at the top of the tube. Because PRF is rich in leukocytes, it is also known as leukocyte- and platelet-rich fibrin (L-PRF). The two most common L-PRF preparation protocols are centrifugation at 3000 rpm for 10 min proposed by Choukroun and 2700 rpm for 12 min proposed by Shahram.^16,17^ Advanced PRF (A-PRF) is obtained by reducing the centrifugation speed and centrifugation time at 1500 rpm for 10 min to achieve a more uniform distribution of leukocytes and platelets in the PRF,^17^ which can also be obtained by centrifugation at 1300 rpm for 14 min.^18^ Pure PRF (P-PRF) or leukocyte-poor PRF can only be obtained by processing whole blood using a commercial FibriNet^®^ Platelet-Rich Fibrin Membrane (PRFM) kit (Cat. No. 510359; Royal Biologics; Hackensack, NJ, USA), which produces a leukocyte-free PRF.^7^ Another method to obtain PRF is to centrifuge whole blood in a titanium tube preferably at 3500 rpm for 15 min, called titanium-prepared PRF (T-PRF).^19^ One advantage to using titanium tubes is that T-PRF is free of silica contaminants, which can be found in PRF prepared in glass tubes and can affect patients receiving PRF.^19^ Horizontal PRF (H-PRF) is a new kind of PRF prepared by horizontal centrifugation at 700 g for 8 min.^20^ Since common PRF contains a strong fibrin matrix and is in the form of an active gel, it is usually compressed into a membrane for use. In 2015, whole blood was collected in hydrophobic plastic tubes with reduced centrifugal force and time at 60 g for 3 min to obtain injectable PRF in liquid form.^21^

#### 3. Concentrated growth factor (CGF)

CGF is a modified form of PRF that contains higher levels of growth factors and platelets and a denser fibrin matrix.^22^ To obtain CGF, whole blood is subjected to repeated centrifugation at acceleration and deceleration rates to activate alpha particles in platelets, producing higher growth factor concentrations.^22^ According to Sacco’s protocol, whole blood was collected without anticoagulant and centrifuged using a variable speed centrifuge (MEDIFUGE™, Silfradent, Sofia, Italy) after 30 s of acceleration, 2700 rpm for 2 min, 2400 rpm for 4 min, 2700 rpm for 4 min, 3000 rpm for 3 min, and deceleration for 36 s to obtain CGF.^23^ Different forms of CGF can be prepared by centrifuging whole blood in different kinds of centrifuge tubes. A dense gel form of CGF is prepared in centrifuge tubes with rough inner walls; a loose gel form of CGF is prepared from tubes with smooth inner walls, and a liquid form of CGF is prepared by adding anticoagulant to whole blood prior to centrifugation.

### B. Properties of PCs

PRP is low in fibrin content, making growth factors trapped in the fibrin reach their peak concentrations on the first day.^24^ PRP-derived growth factors are available and active in the early stages of regeneration and reconstruction.^24^ The rapid release of active factors means that PRP is unsuitable for complex and long-term regeneration processes. On the other hand, PRF releases growth factors for up to 7 to 10 days and has the potential for use in complex and long-term regeneration contexts. As PRF lacks anticoagulants, the natural cascade of coagulation reactions is not impeded, and cross-contamination associated with anticoagulants is avoided. More importantly, naturally agglutinated PRF forms a strong fibrin matrix network that traps many cytokines and growth factors initially secreted by activated platelets, which are gradually released as fibrin degrades.^25^ This makes PRF attractive for longer-term applications than PRP.^26^ Unlike PRP and PRF, A-PRF forms a more porous fibrous scaffold that contains more neutrophils.^17^ Compared with PRP and PRF, A-PRF releases more growth factors within 10 days.^27^ In addition, horizontal centrifugation not only avoids cell damage during angle centrifugation but also enables free migration of cells, resulting in a uniform distribution of cells in H-PRF with higher platelets and leukocytes and more excellent antibacterial effects.^28^ Among the PCs, CGF is best suited for long-term regeneration contexts, as its relatively higher adhesive and tensile strength (compared to PRP and PRF) provides better protection against hydrolysis of growth factors trapped within its fibrin matrix.^29^ This explains why the release of growth factors from CGF has been shown to last up to 28 days.^29^ CGF also promotes angiogenesis by recruiting CD34-positive stem cells from circulating blood.^30,31^

### C. Mechanisms of PCs for tissue repair

There are various mechanisms by which PCs can facilitate tissue repair. Platelets contain large amounts of alpha granules, major reservoirs of growth factors, and cytokines. Upon activation, platelets secrete transforming growth factor-beta, vascular endothelial growth factor (VEGF), epidermal growth factor, insulin-like growth factor-1, and bone morphogenetic protein, which act on target cells to promote cell proliferation and tissue repair.^9,32^ In addition, various bioactive substances, including serotonin, histamine, and adenosine, are released from dense granules.^33^ Fibrinogen and fibronectin within concentrated platelets form a flexible, progressively degradable protein scaffold that encases platelets, cytokines, and growth factors for gradual release, while the scaffold promotes cell adhesion, expansion, and differentiation properties through cell signaling, providing mechanical support for damaged tissue reconstruction.^31,34^ PCs also contain anti-inflammatory cytokines, including interleukin 1β, interleukin 4, interleukin 6, tumor necrosis factor α, and leukocytes, which play regulatory roles in inflammatory and antibacterial processes (Fig. 2).^24^

### D. Platelet lysate (PL)

PLs, which can be obtained from PCs, are another source of growth factors useful for dental tissue repair and regeneration.^35^ The most common method of isolating PLs is to repeatedly freeze and thaw PCs after 3–5 freeze-thaw cycles of 80/37 °C.^36^ Ultrasonication of platelets at a frequency of 20 kHz for 30 min is an alternative method for the preparation of PL.^37^ Compared to PCs, PLs can be stored frozen until use, but its solution form barely forms a gel spontaneously. Processing liquid PL into gel allows for better performance at the surgical site. The primary method of gelation is the addition of thrombin to activate platelets, and there have also been studies of encapsulating PL into hydrogels or chemically cross-linking it with biomaterials.^38^

### E. Platelet-derived extracellular vesicles (P-EVs)

In 1967, Peter Wolf isolated lipid-rich microparticles (MPs) from platelets by ultracentrifugation and discovered these MPs had procoagulant properties.^39^ Further studies revealed that these MPs are extracellular vesicles released by platelets upon activation (Fig. 1).^40^

PCs and PL are the sources of EVs, which can be isolated by differential ultracentrifugation. PRP is centrifuged at 2200 g for 20 min to collect the platelet pellet. The platelet pellet is resuspended and centrifuged at 4 °C at 4000 g for 10 min, 10 000 g for 30 min, and 100 000 g for 70 min to obtain P-EVs.^41^ Aside from that, centrifuging PL at 500 g for 10 min, 2000 g for 15 min, and 10 000 g for 30 min and then ultracentrifuge the supernatant at 30 000 rpm for 1 h is another method to obtain P-EVs.^42^

Based on the difference in diameter, there are two types of EVs, the first being MPs, also called microvesicles, and the second being platelet exosomes (P-EXOs). Microvesicles range from 100 nm to 1 µm in diameter, express surface markers such as Factor X and thrombospondin, and are formed by “budding” from the platelet membrane. P-EXOs are 40–100 nm in diameter and express the exosome-specific marker transmembrane protein CD63 on their surfaces. When the multivesicular bodies and alpha granules fuse with the platelet plasma membrane, the P-EXOs located therein are released by exocytosis.^43^ P-EVs are critical effectors for clotting with procoagulant capacity 50–100 times higher than that of activated platelets. Phosphatidylserine are adhesion receptors on the vesicle surface that provide binding sites for coagulation factor activation and thrombin production.^44^

**FIG. 3.**
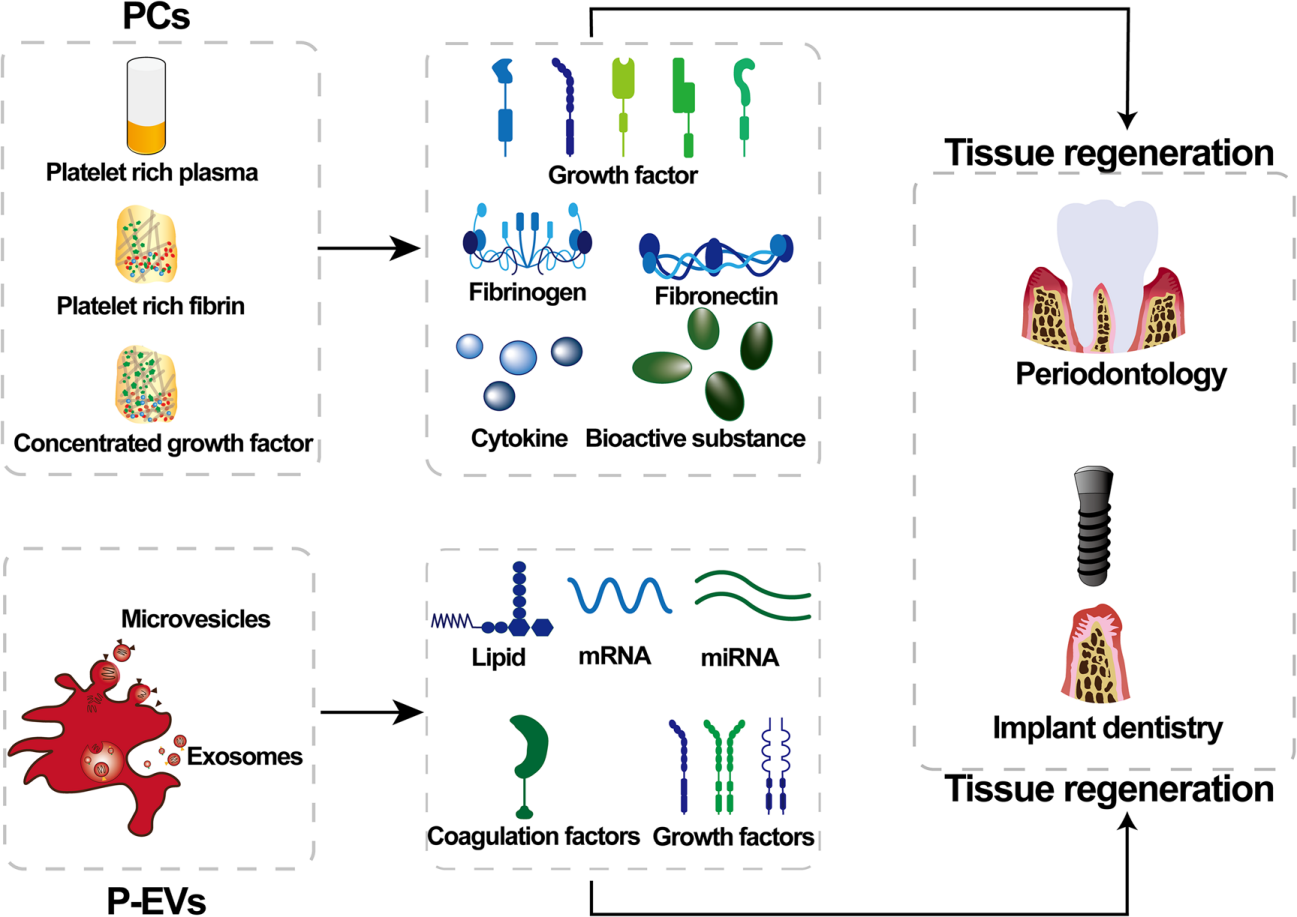
Possible underlying mechanisms of platelet concentrates (PCs) and platelet-derived extracellular vesicles (P-EVs) for tissue regeneration in periodontology and implant dentistry.

Tissue repair studies have demonstrated that P-EVs specifically deliver to their targets bioactive molecules such as growth factors, lipids, coagulation factors, and mRNAs and microRNAs^45^ by explicitly recognizing membrane surface receptor-ligand proteins. These receptors mediate fusion with target cell membranes to regulate the translation of corresponding proteins, improve the physiological function of the target cells, and thereby promote tissue reconstruction.^46^ P-EVs stimulate endothelial cell proliferation and migration through VEGF, basic fibroblast growth factor (bFGF), platelet-derived growth factor (PDGF), and lipids to promote angiogenesis and significantly accelerated peri-implant angiogenesis was observed when P-EVs were filled around the implants.^47^ During hard tissue regeneration, especially osteogenesis, P-EVs enhance proliferation by increasing the incorporation of thymidine by osteoblasts, accelerating the mitogenic response of trabecular cells and promoting bone tissue mineralization and regeneration.^48^ P-EVs isolated from PL expressing bFGF, VEFG, and TGB-β, which are associated with bone healing and bone regeneration with a role in regulating osteoblast migration, proliferation, and differentiation.^42^ Concurrent findings suggest that miRNAs delivered by P-EVs can be involved in the transcription of intracellular signals and phosphorylation of tyrosine by regulating the cAMP, cGMP-PKG, and Rap1 signaling pathway, which, in turn, regulate osteoblast differentiation^49^ (Fig. 3).

In addition, to further improve the bioactivity of P-EVs, their presentation to recipient cells, and their ability to target binding at cell type-specific and tissue-specific levels, some studies have developed engineering P-EVs with the main engineering approaches being modifications of the membranes of extracellular vesicles. Platelet-mimetic EVs were fabricated by fusing the membranes of EVs with platelet membranes by extrusion. This engineering strategy offers an opportunity to design other targeted EVs fused with platelet membranes for therapeutic angiogenesis.^50^ The same team also introduced the platelet membrane-modified EVs based on the membrane fusion method to mimic the binding ability of platelets to monocytes. The new targeted delivery method of EVs to monocytes has the potential to improve immunoregulatory therapy.^51^

### F. Potential for clinical applications

Currently, most clinical use PCs are prepared by simple centrifugation after fresh blood is drawn at the time of application. Although PL can be stored at 4 °C for 14 days and the clinical effect of PL is not worse than PC in clinical treatment, PL needs to be prepared by tedious freezing/thawing or ultrasound method.^52^ The complexity of the preparation process limits the application of PL in oral tissue regeneration to some extent. P-EVs are obtained by differentiated ultracentrifugation of PCs or PL. Although P-EVs’ clinical treatment is not as widespread as PC and PL, P-EVs as endogenous bio carriers can circulate to all human chambers and even the blood–brain barrier. Additionally, engineering P-EVs is a new approach to enhance the potential of P-EVs in biomedical applications such as targeted therapy toward angiogenesis.

## III. CLINICAL APPLICATIONS OF PCs

### A. Hard tissue regeneration

#### 1. Sinus floor elevation

Maxillary sinus floor elevation is a dental surgical procedure that increases the amount of bone in the posterior maxilla. This procedure is done in preparation for, or in conjunction with, installation of dental implants because of bone atrophy or poor bone quality. Maxillary sinus floor augmentation is created by elevating the sinus membrane from the underlying sinus wall and by placing bone grafts to obtain sufficient bone volume to support the dental implants. As technology has evolved, various fillers have been used in order to ensure adequate new bone formation. For many years, autogenous bone grafts were considered the gold standard, but the grafting of autogenous bone inevitably caused damage to the donor site. This often-negative outcome has prompted the use of PCs to simplify the surgical procedure and avoid some complications.

Presently, there is still controversy about whether PRP alone can promote bone regeneration during sinus floor elevation. Some studies have concluded that no benefit was found with the addition of PRP to maxillary sinus floor elevation. However, it has also been shown that using PRP alone finds higher bone augmentation in sinus lifts.^53,54^ Aside from that, PRP can speed up healing after surgery and minimize complications associated with residual graft material.^55^ When PRP is used in combination with autologous bone or bone-graft material, it not only shortens the healing time but also accelerates bone regeneration through vascularization and enhances bone formation in the elevated maxillary sinus floor.^56–59^ Several studies have shown that the use of PRF or CGF alone, without mixing with bone material as a maxillary sinus lift-filling material, and immediate implant placement, can promote new bone formation and produce dense bone-like tissue.^60–66^ When implant surgery is performed immediately after maxillary sinus lift, combining PCs with bone material increases the bone volume at the implant margin and improves the survival and long-term stability of the implant.^22,67,68^ PRF and CGF can also provide protection for the sinus membrane during the use of bone chisels. Even in the case of sinus membrane perforation, the fibrin matrix can help with wound closure^69–71^ (Table I).

**TABLE I.** Applications of PCs and P-EVs in oral hard tissue regeneration.

Material	Comparisons	Effect of PCs	Reference
Maxillary sinus floor elevation			
PRP	PRP	Leads to steady increase.	53
	PRP vs alloplastic graft material		54
	P-PRP + freeze-dried bone allograft (FDBA) vs FDBA	Shortens healing time.	55
	L-PRP + composite bone graft	When combined with PRP, new bone is formed.	56
	PRP + autologous bone grafts vs autologous bone grafts		57
	PRP + iliac crest bone vs iliac crest bone		58
	L-PRF + hydroxyapatite		59
PRF	PRF	Leads to endosinus bone gain.	60–63
	PRF + deproteinized bovine bone mineral	Increases vertical bone height and stabilization.	22, 68
	L-PRF	Repair maxillary sinus membrane perforation.	69, 70
	A-PRF vs collagen membrane		71
CGF	CGF	Induces new bone formation under the elevated sinus membrane with vertical bone gain.	64–66
	CGF + grafted with allograft vs grafted with allograft	Obtains vertical bone height stabilization.	67
Alveolar ridge preservation			
PRP	PRP	Reduces inflammation, promotes soft tissue healing.	73, 74
PRF	PRF vs natural healing	Promotes the healing of soft and bone tissues. reduces pain and discomfort.	76–78
	PRF vs bone allografts		79
CGF	CGF vs natural healing	Reduces vertical and horizontal bone resorption and promotes new bone formation.	80–82
Guided bone regeneration (GBR)			
PRP	PRP	Beneficial in the early healing phase of soft tissue wounds. No significant effect on bone height changes.	85–89
PRF	PRF	Improves implant stability, implant survival and marginal bone level.	90, 91
	PRF + autogenous and xenogenous grafts + collagen membrane		94
	PRF + autogenous bone + bovine inorganic bone graft		95
CGF	CGF	Promotes horizontal and vertical bone regeneration.	31
	CGF vs collagen membrane		96
	CGF + mineralized collagen vs mineralized collagen		97
	CGF + bone graft matrix vs bone-shell technique		98
P-EVs	P-EVs	Promotes osteogenic differentiation.	99, 100
Periodontal intrabony defects repair			
PRP	PRP + demineralized freeze-dried bone allograft vs demineralized freeze-dried bone allograft	Increases CAL and improves gingival recession, but no effect on the gain of hard tissue filling or new hard tissue formation.	101
	PRP + bone mineral + GTR (guided tissue regeneration) vs bone mineral + GTR		102
	PRP + β-TCP + GTR vs β-TCP + GTR		103
	PRP + demineralized freeze-dried bone allograft vs demineralized freeze-dried bone allograft		104
PRF	PRF vs OFD	Reduces the bone defect depth and increases bone fill.	108
	PRP + OFD vs OFD		109
	PRF+ OFD vs autogenous bone graft + OFD		110
	PRF+ freeze-dried bone allograft vs freeze-dried bone allograft	Combination of PRF is significantly beneficial to clinical defect depth reduction and defect filling.	111
	PRF vs PRF + Bovine porous bone mineral		112
	PRF + bovine porous bone mineral + GTR vs bovine porous bone mineral + GTR		113
CGF	CGF	Reduces PD, increases CAL and bone level height, and fills bone defects.	115
	CGF vs CGF +demineralized freeze-dried bone allograft		116
	CGF + bovine bone mineral vs bovine bone mineral	Obtains better bone defect repair results.	117
	Flap surgery vs flap surgery + CGF vs flap surgery + Bio-Oss vs flap surgery + CGF + Bio-Oss		118

#### 2. Alveolar ridge preservation

**FIG. 4.**
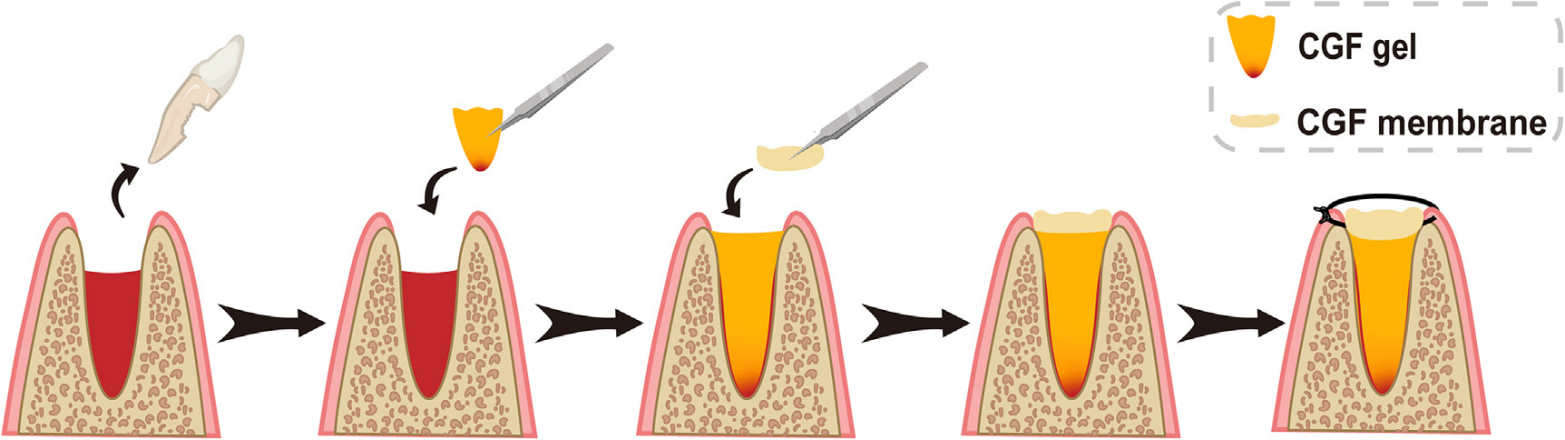
Schematic diagram of the surgical process of CGF applied to alveolar ridge preservation after tooth extraction (sagittal view).

Alveolar ridge preservation is the process of filling the post-extraction socket with some bone material or scaffold to reduce bone resorption after extraction, preserve good alveolar ridge structure and soft tissue morphology, and ensure sufficient space for implant placement.^72^ After tooth extraction, filling with PRP can relieve pain and swelling, prevent food build-up, reduce oral odor, and to some extent promote the healing of hard and soft tissues.^73,74^ However, PRP has a low mechanical strength and is not easy to use it to fill sockets.^75^ PRF, on the other hand, possesses much better mechanical strength, and when used alone, it not only accelerates soft tissue wound healing but also reduces the resorption of alveolar ridge bone height and width. PRF also enhances bone density, as evidenced by both clinical results and radiographic measurements.^76–79^ CGF application following posterior tooth extraction may reduce vertical and horizontal bone resorption and promote new bone formation (Fig. 4).^80^ However, the use of CGF alone for alveolar ridge preservation is currently rare, and more data are needed to demonstrate that CGF used to fill extraction sockets significantly changes the quality of bone tissue^81,82^ (Table I).

#### 3. Guided bone regeneration (GBR)

Osseointegration after implant placement is the basis for successful implantation. Osseointegration after implant placement is the basis for successful implantation. One of the key factors for attaining osseointegration is the presence of an adequate osseous volume. The GBR technique is commonly used for bone augmentation in implant dentistry today. The critical issue with the GBR technique is the placement of an occlusive membrane that will prevent connective tissue cells from colonizing the defect and provide enough space to allow for bone regeneration of the entire defect volume.^83,84^

How do PC products contribute to this whole process? Only a transient and slight beneficial effect on early osseointegration was observed after the implant surface was treated with activated PRP impregnation prior to implant placement. During implantation, PRP liquid mixed with bone material and applied to the implant socket was less important for long-term survival of the implant.^85–89^ When GBR is used for dental implants, PRF and CGF can be compressed like a membrane for mineralizing blood clots while also preventing excess cells from entering the area of osseointegration and providing space for the migration of osteoblasts and angiogenic cells.^90,91^ The use of PRF and CGF improves the stability of the implant, provides for rapid osseointegration, and enhances the integration of the implant and the marginal bone volume around the implant.^31,92–98^

P-EVs are intrinsically osteoinductive and can induce osteogenic differentiation of stem cells in the absence of any external induction.^99^ Some have attempted to coat the surface of titanium implants with P-EVs, which improves the biocompatibility of titanium. It is gradually released over time, in the hope of improving the osteogenic properties of titanium implants^100^ (Table I).

#### 4. Periodontal intrabony defects repair

Periodontal disease is a chronic inflammatory condition in which horizontal and vertical resorption of the alveolar bone results in varying degrees of intra-periodontal bone defects. These can ultimately result in loss of tooth support structures and loosening of teeth. Treatment of periodontal bone defects requires enhanced regeneration of the defective bone to maintain normal occlusal function. Currently, open flap debridement (OFD), GBR, and the use of grafts such as enamel matrix derivatives or bone material are currently used for periodontal intraosseous defects (IBDs) in order to promote regeneration.

For the repair of dental intraosseous defects, it is important to gain clinical attachment level (CAL) and gingival marginal level (GML) in addition to probing depth (PD) reduction. However, a reduction in the depth of the intraosseous defect (IBD) and an increase in bone fill (BF) are more important. For regeneration of intraosseous defects, at least four to six weeks are required to guide bone regeneration in order to block the epithelium. PRP is sometimes used to treat periodontal intraosseous defects. However, due to the rapid degradation of PRP, its beneficial effect on hard tissues is limited when it is used alone to treat periodontal intraosseous defects. Combining grafts with PRP or applying PRP in an OFD arrangement results in better soft tissue performance such as in the case of CAL gain and PD reduction in periodontal bone defects but is not better for hard tissue filling or new hard tissue formation.^101–105^

Studies show that in addition to the role of growth factors in periodontal bone defects, fibronectin in PRF also promotes the formation of new blood vessels and the conversion of undifferentiated mesenchymal cells in the blood into osteoblasts.^106,107^ Not only does the combined use of PRF achieve better CAL gain and PD reduction compared to OFD surgery alone, a superior reduction in the depth of the intraosseous defect is achieved, and an increase in bone filling is observed both clinically and radiologically.^108–110^ When PRF is used in combination with bone substitutes, better clinical outcomes result than with PRF alone.^111–113^ CGF still has a pro-osteogenic effect on human periodontal ligament cells in an inflammatory microenvironment that mimics periodontitis.^114^ When used alone, CGF reduces PD, enhances CAL, and good bone regeneration is achieved.^115,116^ This effect is even more pronounced when used in combination with bone graft material^117,118^ (Table I).

### B. Soft tissue regeneration

#### 1. Treatment of gingival recession

Gingival recession is one of the main symptoms of periodontitis, which can lead to extensive root exposure and tooth sensitivity. The main root-coverage procedures currently used to treat gingival recession are coronally advanced flap (CAF) and subepithelial connective tissue grafts (SCTGs).

In the case of gingival recession, attempts have been made to use PRP and PRF alone or combine with CAF or SCTG in Miller classes I and II gingival recessions. The application of PRP and PRF promotes early soft tissue healing and improves root coverage, depth of the gingival recession, and CAL.^119–125^ For gingival recession applications, PRF has advantages over PRP in liquid or gel form, as PRF can be compressed into a membrane and used alone as a barrier membrane to guide tissue regeneration to cover the surface of the exposed tooth root. Gingival fibroblasts are a significant component of gingival connective tissue. PRP and PRF promote the migration and proliferation of gingival fibroblasts and the expression of type I collagen by human gingival fibroblasts.^126^ However, some studies have concluded that PRP and PRF have very limited efficacy for improving the width of keratinized tissue, and only CGF films can significantly improve keratinized tissue.^127–129^

P-EVs are biocompatible *in vitro* systems, promote regeneration of gingival keratinocytes and granulocytes, and express genes related to gingival remodeling. P-EVs can also be combined with biomaterials such as hyaluronic acid to help maintain their regenerative effect while research seeks more applicable treatments^130^ (Table II).

**TABLE II.** Applications of PCs and P-EVs in oral soft tissue regeneration.

Material		Comparisons	Effect of PCs	References
Treatment of gingival recession				
PRP	PRP + CTG vs CTG		Additional application of PRP reduces vertical recession depth and PD, improves CAL.	119
	PRP + CAF			120
	PRP + CTG			121
PRF	PRF + CAF		Significantly improves PD and CAL, increases gingival thickness and improves root coverage.	122
	PRF + CAF vs CAF			123, 124
	PRF + CAF vs CAF + CTG			125
CGF	CGF vs CTG + CAF		Increases the width of keratinized tissue	127
	CGF + CAF vs CAF			128
	CGF + CAF vs PRF + CAF			129
P-EVs	P-EVs		Promotes regeneration of gingival keratinocytes and granulocytes, enhances gene expression during gingival healing.	130
Periodontal soft tissue regeneration				
PRP	PRP + OFD		Combining with PRP increases CAL, reduces PD.	131, 132
	PRP + bone mineral + GTR vs bone mineral + GTR			102
	PRP + β-TCP + GTR vs β-TCP + GTR			103
	PRP vs PRP + collagen sponge			134
PRF	PRF + OFD vs OFD		Increases CAL, reduces PD.	135
	PRF + demineralized freeze-dried bone allograft vs demineralized freeze-dried bone allograft			136
	PRF gel vs PRF gel + PRF membrane vs OFD		PRF alone promotes PD reduction and CAL gain.	137
	PRF vs OFD			
				138
CGF	CGF		CGF alone promotes PD reduction, CAL gain.	115
	CGF vs CGF + demineralized freeze-dried bone allograft			
				116
P-EVs	P-EVs		Promotes angiogenesis, epithelial formation and wound healing.	13, 142
Soft tissue augmentation around implants				
PRP	PRP		Promotes wound healing after implantation.	89, 144
PRF	PRF		Increases peri-implant soft tissue thickness and keratinized tissue width.	145
	PRF vs CTG			146
	PRF vs OFD			147
	PRF vs free gingival graft			148
PRF	PRF		Almost negligible effect on thickening of the tissue around the neck implant.	93, 149

#### 2. Periodontal soft tissue regeneration

Application of PCs provide various growth factors when used for regenerating periodontal soft tissues in the treatment of periodontal disease. PDGF can recruit progenitor cells from periodontal tissues, promote cell proliferation and migration and play an important role in periodontal healing. bFGF effectively promotes the proliferation and migration of periodontal membrane fibroblasts. VEGF recruits progenitor cells to periodontal wounds, promotes endothelial cell proliferation, and contributes to angiogenesis and hematopoietic reconstitution of periodontal tissue. Angiogenesis in this context plays a key role in mitigating the pathological process and in the regeneration of periodontal tissue. Several studies have found that beta-lysin and neutrophil activating protein 2 in PCs are anti-microbial.^22^

In restoring periodontal soft tissues, the use of PRP after open flap debridement (OFD) produced a higher CAL than OFD alone.^131,132^ The outcome is even better when combined with a guided tissue regeneration membrane.^133^ When applied in combination with bone material to treat periodontal defects, it has shown a positive clinical impact in terms of soft tissue defects in the periodontium.^102,103,134^ In treating periodontal soft tissue in combination with OFD, guided tissue regeneration (GTR), and bone graft material, PRF and CGF enhance attachment and reduce the depth of clinically treated periodontal pockets.^116,135,136^ PD reduction and CAL gain were also obtained with PRF or CGF alone.^115,137,138^ However, PRF and CGF are more likely to fill the defect in the form of a membrane, which not only stabilizes the blood clot for periodontal regeneration, but also acts as a competitive barrier, one that both blocks epithelial cell and facilitates the growth of the periodontal ligament into the defective area.^139^ More importantly, PRF and CGF have a significant regenerative effect on fixing defects within periodontal bone, providing attachment sites for new periodontal ligament fibers.^140^

Platelet lysate- (PL)-based materials can promote soft tissue healing in periodontal applications,^38,141^ and P-EVs are important effectors of PL activity.^42^ P-EVs facilitate wound healing by promoting the proliferation and migration of fibroblasts, which, in turn, stimulates re-epithelialization and angiogenesis. This suggests that P-EVs may be used in the clinic to promote periodontal tissue healing and regeneration^13,142^ (Table II).

#### 3. Soft tissue augmentation around implants

Increasing the width of the peri-implant keratinized tissue and mucosal thickness can effectively improve the functional, esthetic, and biological outcome parameters after implant placement.^143^ Currently used surgical procedures for increasing the width of keratinized gingiva and mucosal thickness include apically positioned flap with vestibuloplasty or simultaneous use of autologous tissue or collagen matrix.

**FIG. 5.**
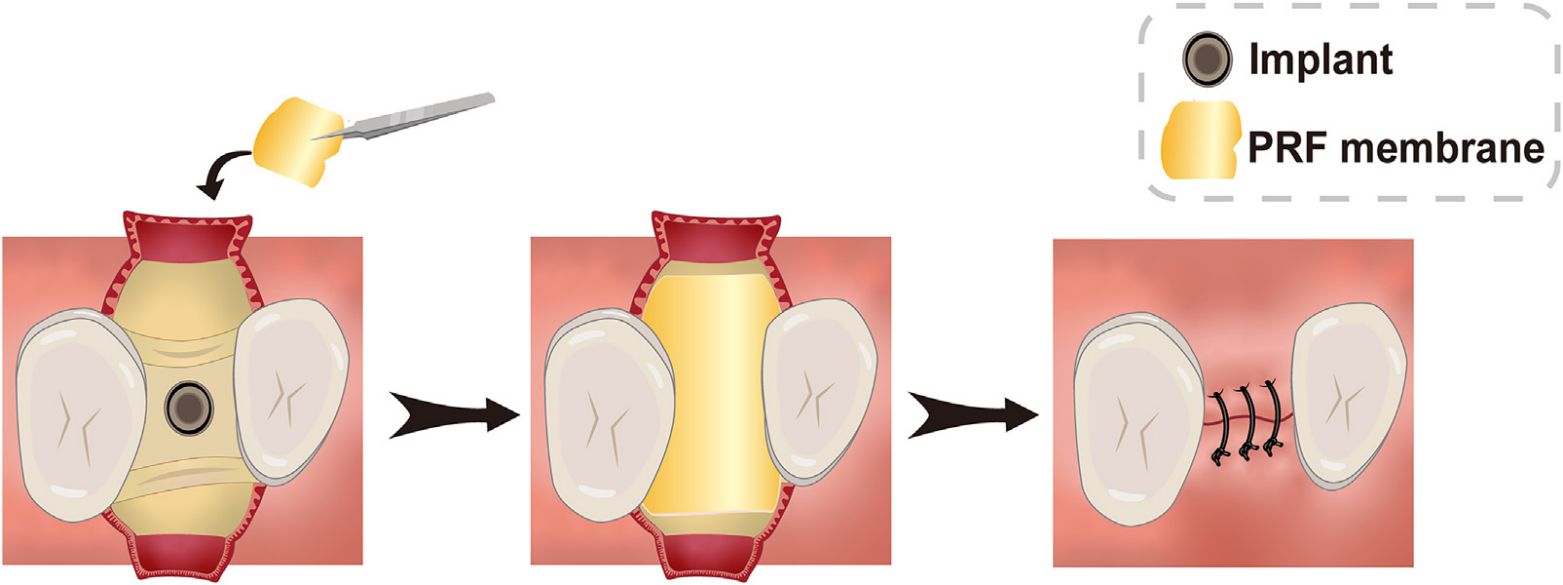
Schematic illustration of the surgical procedure for thickening of peri-implant mucosa with PRF membrane (occlusal view).

PRP facilitates early wound healing in soft tissues when used in implant surgery, but it is not effective for peri-implant soft tissue regeneration.^89,144^ Implant placement combined with PRF results in an increase in the thickness of the soft tissue around the implant (Fig. 5).^145–148^ However, the ability of PRF to increase the peri-implant tissue remains controversial.^93,149^ Although CGF has a strong effect on wound healing, most current studies have focused on the effectiveness of CGF in promoting bone regeneration around implants. More in-depth studies are needed to determine whether CGF has demonstrated capacity to augment soft tissues around implants (Table II).

## IV. CONCLUSION AND PERSPECTIVES

PCs and P-EVs are safe, reliable products for bone augmentation and soft tissue augmentation. Originating from standard autologous blood collection procedures, they are easy to prepare and have rich and extensive applications. However, there are still no uniform standards in place for the preparation of PCs and P-EVs. Nor, is it completely understood yet how a patient’s systemic health affects their active ingredients. This latter gap in knowledge may partly explain the reported variability in their use for implant surgery and periodontal tissue regeneration. However, we still believe that they have potential and that there is an urgent need to develop uniform industry standards and clinical guidelines for their clinical use aimed to enhance tissue repair. Large randomized, controlled clinical trials in which patients’ systemic health status is documented are warranted to rigorously evaluate the potential of uniformly prepared PC products in regenerative periodontological and implant dentistry applications.

## Data Availability

The data that support the findings of this study are available from the corresponding author upon reasonable request.
